# Analysis of influencing factors and interaction effects on stroke recurrence in patients with middle cerebral artery occlusion treated with mechanical thrombectomy

**DOI:** 10.3389/fneur.2025.1580950

**Published:** 2025-08-21

**Authors:** Guoliang Li, Zhen Feng, Huiyan Zhang, Yongzhou Zou, Hong Xv, Shunfu Jiang

**Affiliations:** Department of Neurology, Jingdezhen First People's Hospital, Jingdezhen, China

**Keywords:** middle cerebral artery occlusion, mechanical bolt removal, stroke recurrence, interaction effect, MCA occlusion

## Abstract

**Background:**

Stroke recurrence is an important factor affecting the prognosis of mechanical thrombectomy in patients with middle cerebral artery (MCA) occlusion. This study aims to construct a model for evaluating the degree of stroke recurrence and conduct binary and ternary interaction analysis.

**Method:**

We conducted a retrospective analysis of the clinical data of stroke recurrence patients, collecting demographic data, clinical characteristics, treatment factors, and biochemical indicators. Use XGBoost and RF models to screen features that contribute significantly to the degree of recurrence, and evaluate model performance through indicators such as ROC curve, F1 score, accuracy, and recall. Construct a stroke recurrence evaluation model based on the common features selected from these two models. Use the Andersson model to analyze the binary interaction between the model and other factors. Further analyze the three-way interaction between the model and other factors.

**Result:**

Both XGBoost and RF models perform well. In the multivariate logistic regression analysis, the recurrence model showed that age, smoking history, and infarct size had a significant impact on the degree of stroke recurrence (OR = 1.006, 1.214, 1.167, all *p* < 0.05), and the constructed recurrence model had a significant effect on the degree of stroke recurrence (OR = 1.346, *p* = 0.047). Through binary interaction analysis, it was found that there was a significant antagonistic effect between the recurrence model and age, smoking history, and infarct size. Triple interaction analysis showed that the synergistic effect of the recurrence model with age and smoking history was significant, and the synergistic effect of the recurrence model with smoking history and infarct size was also significant.

**Conclusion:**

Age, smoking history, and infarct size are important influencing factors on the degree of stroke recurrence in MCA occlusion patients after mechanical thrombectomy treatment. The recurrence model performs differently in different patient populations, and the interaction with age, smoking history, and infarct size is of great significance for evaluating the degree of stroke recurrence.

## 1 Introduction

Middle cerebral artery (MCA) occlusion is a common type of acute ischemic stroke (1). The MCA occlusion is one of the blood vessels that supply the main areas of the cerebral hemisphere, responsible for providing blood to the frontal, parietal, temporal lobes, as well as some basal ganglia and subcortical structures of the brain (2, 3). When the middle cerebral artery is blocked, ischemic damage occurs to the brain tissue in the supplied area, leading to corresponding clinical symptoms such as limb hemiplegia, language disorders, and sensory loss. Mechanical thrombectomy (MT) is an important treatment method for middle cerebral artery occlusion (4, 5). The basic principle is to directly remove thrombi from the blood vessels through mechanical devices, thereby restoring cerebral blood flow, reducing or avoiding ischemic injury (6, 7). Although it can significantly improve the clinical outcomes of patients, the existence of recurrence risk makes evaluating stroke recurrence an urgent problem in clinical decision-making. Therefore, building an accurate model to assess the risk and degree of stroke recurrence in MCA occlusion patients undergoing mechanical thrombectomy therapy is of great significance for clinical intervention and personalized treatment.

Stroke recurrence refers to the occurrence of new cerebrovascular events in patients after the stroke treatment, which further exacerbates neurological dysfunction (8). Its occurrence is closely related not only to the severity of the primary stroke, the patient’s underlying disease, and lifestyle factors, but also to multiple factors during the treatment process such as treatment window, thrombectomy time, thrombus type, etc. For MCA occlusion patients, the occurrence of recurrent stroke often means that cerebral blood flow is interrupted again, leading to further deterioration of already damaged brain tissue and affecting the patient’s functional recovery and quality of life (9). In recent years, although significant progress has been made in the treatment strategy of MCA occlusion using mechanical thrombectomy technology, evaluating stroke recurrence remains a complex and challenging task.

Existing research has focused more on clinical prognostic indicators and treatment outcomes for stroke patients (10, 11), and there have also been studies exploring the related influencing factors of mechanical thrombectomy followed by occlusion (12). However, there has been no further classification of occlusion degree, and no analysis of the multifactorial interaction of stroke recurrence. This study fills these gaps by retrospectively analyzing clinical data of MCA occlusion patients, constructing a machine learning algorithm based stroke recurrence evaluation model, and exploring in depth the impact of the interaction between the recurrence model and different factors on stroke recurrence, providing more accurate recurrence evaluation basis for clinical practice.

## 2 Materials and methods

### 2.1 Research object

The study subjects were patients with middle cerebral artery occlusion who underwent mechanical thrombectomy and experienced stroke recurrence within 90 days. All patients’ clinical data were sourced from our hospital, and the study period was from January 2019 to January 2024. Inclusion criteria: 1. Age ≥ 18 years; 2. Diagnosed with acute ischemic stroke due to MCA occlusion and underwent mechanical thrombectomy; 3. Complete clinical data, including demographic data, clinical characteristics, treatment factors, and biochemical indicators; 4. Complete follow-up data after treatment, with a 90-day follow-up. Exclusion criteria: 1. Severe comorbidities, such as malignant tumors, end-stage renal disease, etc.; 2. Severe liver and kidney dysfunction; 3. Stroke recurrence not occurring within 90 days; 4. Did not receive mechanical thrombectomy before stroke recurrence; 5. Severe mental illness or poor compliance; 6. Incomplete data or lack of follow-up.

### 2.2 Thrombectomy method

Local anesthesia is used, and the patient is placed in the supine position. A right femoral artery puncture is performed using the Seldinger technique. Digital subtraction angiography (DSA) is conducted to precisely assess the location of the occluded vessel. A guidewire is introduced, and a microcatheter is advanced under the guidance of the guidewire to the distal end of the occluded artery. A 4 mm × 20 mm Solitaire FR stent is then deployed. After releasing the stent for 2–5 min, both the stent and microcatheter are withdrawn. The procedure ends when DSA shows a good contrast of the middle cerebral artery.

### 2.3 Data collection

This study is an observational study, collecting the clinical data of patients with MCA occlusion, including demographic characteristics (such as age, gender, BMI, etc.), medical history (such as stroke history, history of cardiovascular disease, history of diabetes, history of hyperlipidemia, history of smoking and drinking, etc.), clinical symptoms (such as hemiplegia, aphasia, visual impairment, etc.), stroke severity assessment (such as NIHSS score, infarct area and infarct site), and treatment related factors (such as treatment window period, intraoperative blood loss, embolectomy time, puncture site selection, catheter insertion method and embolus type). We also collected biochemical indicators of patients upon admission, including white blood cell count (WBC, x 10 ^ 9/L), neutrophil percentage, platelet count (x 10 ^ 9/L), C-reactive protein (CRP, mg/L), interleukin-6 (IL-6, pg/mL), interleukin-10 (IL-10, pg/mL), total bilirubin (mg/dL), and lactate dehydrogenase (LDH, U/L).

### 2.4 Classification of stroke recurrence degree

The diagnostic basis for stroke recurrence is a comprehensive evaluation of clinical manifestations and imaging examinations. According to the degree of stroke recurrence, patients are divided into the following three groups: Mild recurrence group: The new infarction area is usually small, located at the edge of the previous infarction zone, appearing as a small area of low density (CT) or high signal (MRI diffusion-weighted imaging, DWI). The infarct volume is less than 1 cm^3^, and the infarct diameter is less than 1 cm. The NIHSS score ranges from 0 to 5. Moderate recurrence group: The infarction area is larger, ranging from 1 to 3 cm in low density (CT) or high signal (DWI), with infarct volume between 1 and 3 cm^3^. It involves the cerebral cortex, basal ganglia, or small portions of white matter, but does not affect the main vascular territories. The NIHSS score ranges from 6 to 15. Severe recurrence group: The infarction area is larger than 3 cm, involving the entire cerebral lobe, brainstem, or basal ganglia, including extensive brain regions supplied by major vessels such as the middle cerebral artery (MCA), anterior cerebral artery (ACA), or posterior cerebral artery (PCA). The imaging shows extensive low density (CT) or high signal (MRI), with infarct volume greater than 3 cm^3^, and the NIHSS score is greater than or equal to 16. If the NIHSS score and imaging findings do not match, the higher value should be taken as the reference.

### 2.5 Statistical analysis

Due to the small number of patients with severe recurrence, we coded both moderate and severe recurrence as 1, and mild recurrence as 0, and analyzed the degree of stroke recurrence as the dependent variable. The data was divided into training and test set using a 7:3 ratio. First, we processed the training set and used the train function from the caret package to perform hyperparameter tuning for the XGBoost model. We applied 10-fold cross-validation and combined multiple hyperparameter combinations for grid search to optimize model performance. By selecting the best hyperparameters, we constructed the final XGBoost model. After training, we made predictions on the test set, using the predicted probabilities for binary classification and converting them to class labels based on a threshold of 0.5. We then evaluated the model’s performance using a confusion matrix to obtain accuracy, precision, recall, and F1 score. We applied bootstrap resampling (*n* = 1,000) to estimate performance metrics and calculate the confidence intervals. Finally, we used SHAP values to analyze the model’s interpretability and created a beeswarm plot to reveal the contribution of each feature to the prediction results. For the random forest model, similar to the XGBoost model, we performed 10-fold cross-validation on the training set for model training and hyperparameter tuning. After training, we made predictions on the test set using the trained model, and similarly constructed a confusion matrix to obtain accuracy, precision, recall, and F1 score. After completing model training and evaluation, we used the importance function from the randomForest package to retrieve the GINI index of each feature and created a feature importance plot to display the contribution of each feature to the model’s predictions. The performance of both models was evaluated using ROC curves. Based on the feature importance values, we selected the top 5 common features that contribute to evaluating stroke recurrence severity from both models, and built the stroke recurrence model using the following formula:

Recurrence model = Factor _[1]_*Coef _[1]_+ Factor _[2]_*Coef _[2]_+ Factor _[3]_*Coef _[3]_+ … Factor_[n]_*Coef_[n]_.

Among them, Coef is the sum of the standardized SHAP value and GINI index of the variable.

Patients with a recurrence model score greater than the median of the recurrence model are coded as 1, otherwise coded as 0; Patients with age greater than the median are coded as 1, otherwise coded as 0. Using the Andersson model to analyze the binary interaction between the recurrence model and other factors, the Andersson model has three indicators, namely RERI, AP, and S (13). If RERI and AP are less than 0 and S is less than 1, it is considered an antagonistic effect. If RERI and AP are greater than 0 and S is greater than 1, it is considered a synergistic effect. This effect only occurs when the 95% confidence interval of RERI and AP does not include 0, the 95% confidence interval of S only holds when it does not include 1. As long as one of these three indicators meets the above conditions, it is considered that there is an interaction (synergy or antagonism). We used a multivariable logistic regression model to analyze the impact of the recurrence model and significant factors from univariate analysis on the degree of stroke recurrence. Significant factors from the multivariable logistic regression analysis were selected as independent variables, with stroke recurrence severity (mild coded as 0, moderate and severe coded as 1) as the dependent variable, ternary interaction analysis was performed to evaluate the distinguishing effect of the recurrence model in combination with multiple factors on stroke recurrence severity.

## 3 Results

### 3.1 Differences in demographic, clinical characteristics, and treatment-related factors among patients with different degrees of stroke recurrence

The results showed that in terms of age, patients in the severe recurrence group were generally older; In terms of gender distribution, male patients account for the majority, but there is no significant difference between the groups. BMI, The history of diabetes and alcohol consumption were not significantly different among the groups, while the history of cardiovascular disease and smoking accounted for a higher proportion in the severe relapse group and were significant. The severity of aphasia and hemiplegia increases with the severity of recurrence, with a higher proportion of severe aphasia patients in the severe recurrence group. There is no significant difference in stroke history across the groups, but infarct size differs among them, with larger infarct sizes being more prevalent in the severe recurrence group. Treatment window, thrombectomy time, and intraoperative blood loss also vary among patients with different recurrence degrees, with a higher proportion of severe recurrence patients having longer treatment windows, longer thrombectomy times, and greater intraoperative blood loss. In addition, the types of thrombus vary among patients with different degrees of recurrence. The proportion of the three types of thrombus is similar in patients with severe recurrence, while soft thrombus accounts for a higher proportion in patients with mild and moderate recurrence. In summary, age, cardiovascular disease history, smoking history, aphasia at onset, infarct size, treatment window, intraoperative blood loss, thrombectomy time, and thrombus types differ in their proportions across patients with varying degrees of stroke recurrence (Table 1).

**Table 1:** Differences in demographic, clinical characteristics, and treatment-related factors among patients with different degrees of stroke recurrence.

Variables	All (*n* = 272)	Mild (*n* = 126)	Moderate (*n* = 104)	Severe (*n* = 42)	*p*-value
Age	61 (42–79)	58 (42–79)	62 (42–78)	63 (44–78)	0.0359
Gender					0.08048525
Male	153 (56.25%)	75 (59.52%)	61 (58.65%)	17 (40.48%)	
Female	119 (43.75%)	51 (40.48%)	43 (41.35%)	25 (59.52%)	
BMI	26.1 (18.2–35.0)	26.1 (18.2–35.0)	26.2 (18.3–35.0)	25.6 (18.4–35.0)	0.542
Stroke history					0.3725569
Yes	54 (19.85%)	27 (21.43%)	22 (21.15%)	5 (11.9%)	
No	218 (80.15%)	99 (78.57%)	82 (78.85%)	37 (88.1%)	
Cardiovascular disease history					0.02246609
Yes	51 (18.75%)	29 (23.02%)	11 (10.58%)	11 (26.19%)	
No	221 (81.25%)	97 (76.98%)	93 (89.42%)	31 (73.81%)	
Diabetes history					0.1653374
Yes	42 (15.44%)	14 (11.11%)	19 (18.27%)	9 (21.43%)	
No	230 (84.56%)	112 (88.89%)	85 (81.73%)	33 (78.57%)	
Dyslipidemia					0.05522594
Yes	98 (36.03%)	41 (32.54%)	35 (33.65%)	22 (52.38%)	
No	174 (63.97%)	85 (67.46%)	69 (66.35%)	20 (47.62%)	
Smoking history					0.01773389
Yes	79 (29.04%)	26 (20.63%)	38 (36.54%)	15 (35.71%)	
No	193 (70.96%)	100 (79.37%)	66 (63.46%)	27 (64.29%)	
Alcohol consumption history					0.2653886
Yes	86 (31.62%)	44 (34.92%)	33 (31.73%)	9 (21.43%)	
No	186 (68.38%)	82 (65.08%)	71 (68.27%)	33 (78.57%)	
Hemiparesis					0.06776766
No or Mild	148 (54.41%)	80 (63.49%)	47 (45.19%)	21 (50%)	
Moderate	84 (30.88%)	33 (26.19%)	38 (36.54%)	13 (30.95%)	
Severe	40 (14.71%)	13 (10.32%)	19 (18.27%)	8 (19.05%)	
Aphasia					0.001456267
No or Mild	140 (51.47%)	78 (61.9%)	49 (47.12%)	13 (30.95%)	
Moderate	109 (40.07%)	43 (34.13%)	45 (43.27%)	21 (50%)	
Severe	23 (8.46%)	5 (3.97%)	10 (9.62%)	8 (19.05%)	
Visual disturbances					0.1194414
No or Mild	152 (55.88%)	81 (64.29%)	51 (49.04%)	20 (47.62%)	
Moderate	107 (39.34%)	41 (32.54%)	46 (44.23%)	20 (47.62%)	
Severe	13 (4.78%)	4 (3.17%)	7 (6.73%)	2 (4.76%)	
NIHSS	13 (6–21)	13 (6–21)	14 (6–21)	16 (6–21)	0.0112
Size of the infarct area					0.003650482
Small infarction	31 (11.4%)	20 (15.87%)	5 (4.81%)	6 (14.29%)	
Moderate infarction	149 (54.78%)	75 (59.52%)	51 (49.04%)	23 (54.76%)	
Large infarction	92 (33.82%)	31 (24.6%)	48 (46.15%)	13 (30.95%)	
Side of the infarct area					0.3664327
Left-sided infarction	157 (57.72%)	77 (61.11%)	61 (58.65%)	19 (45.24%)	
Right-sided infarction	97 (35.66%)	42 (33.33%)	37 (35.58%)	18 (42.86%)	
Bilateral infarction	18 (6.62%)	7 (5.56%)	6 (5.77%)	5 (11.9%)	
MCA occlusion site classification					0.2847687
MCA trunk occlusion	59 (21.69%)	25 (19.84%)	21 (20.19%)	13 (30.95%)	
MCA branch occlusion	213 (78.31%)	101 (80.16%)	83 (79.81%)	29 (69.05%)	
Treatment window	5.2 (0.1–10.1)	4.6 (0.1–9.9)	4.9 (0.2–10.1)	6.3 (0.4–9.9)	0.0217
Intraoperative blood loss					0.04334397
Minimal or No hemorrhage (<5 mL)	117 (43.01%)	44 (34.92%)	54 (51.92%)	19 (45.24%)	
Small hemorrhage (5–20 mL)	153 (56.25%)	82 (65.08%)	49 (47.12%)	22 (52.38%)	
Moderate to Large hemorrhage (>20 mL)	2 (0.74%)	0 (0%)	1 (0.96%)	1 (2.38%)	
Thrombectomy duration (min)	57 (42–73)	54 (42–73)	56 (42–73)	62 (43–73)	0.0151
Puncture site selection					0.1699727
Femoral artery puncture	195 (71.69%)	94 (74.6%)	68 (65.38%)	33 (78.57%)	
Radial artery puncture	77 (28.31%)	32 (25.4%)	36 (34.62%)	9 (21.43%)	
Catheter insertion method					0.144166
Unilateral puncture	260 (95.59%)	123 (97.62%)	99 (95.19%)	38 (90.48%)	
Bilateral puncture	12 (4.41%)	3 (2.38%)	5 (4.81%)	4 (9.52%)	
Thrombus type					0.001979872
Soft thrombus	135 (49.63%)	67 (53.17%)	53 (50.96%)	15 (35.71%)	
Hard thrombus	40 (14.71%)	18 (14.29%)	8 (7.69%)	14 (33.33%)	
Mixed thrombus	97 (35.66%)	41 (32.54%)	43 (41.35%)	13 (30.95%)	

### 3.2 Differences in inflammatory response, immune function, and liver function related blood biochemical indicators among patients with different degrees of stroke recurrence at admission

The results showed that the white blood cell count, neutrophil percentage, C-reactive protein, IL-6, and lactate dehydrogenase levels in the severe stroke recurrence group were significantly higher than those in the mild and moderate recurrence groups, indicating that patients in this group had stronger inflammatory reactions and more severe tissue damage upon admission. In addition, IL-10 levels were higher in the severe recurrence group, and there was no significant difference in total bilirubin levels among patients with different degrees of recurrence. Overall, patients with severe stroke recurrence exhibit more significant inflammation and immune responses, but liver function does not show significant differences (Table 2).

**Table 2:** Differences in inflammatory response, immune function, and liver function-related blood biochemical indicators at admission among patients with different degrees of stroke recurrence.

Variables	All (*n* = 272)	Mild (*n* = 126)	Moderate (*n* = 104)	Severe (*n* = 42)	*p*-value
White blood cell count, WBC (×10^9 L)	12.3 (8.4–16.6)	11.8 (8.4–16.6)	12.4 (8.5–16.5)	14.0 (8.6–16.5)	0.0167
Neutrophil percentage (%)	75.7 (69.2–82.4)	75.4 (69.3–82.3)	75.5 (69.2–82.4)	78.1 (69.6–82.2)	0.0237
Platelet (× 10^9/L)	279.6 (174.5–374.3)	276.6 (178.5–369.9)	282.0 (174.5–374.3)	271.0 (176.5–351.1)	0.666
C-Reactive protein (mg/L)	45.1 (17.6–72.1)	42.6 (17.6–71.8)	43.9 (17.9–70.9)	58.4 (21.8–72.1)	0.00831
IL-6 (pg/mL)	238.0 (55.2–423.0)	218.5 (67.6–421.5)	228.8 (57.4–419.2)	323.2 (55.2–423.0)	0.0183
IL-10 (pg/mL)	22.4 (13.9–32.3)	21.5 (13.9–32.3)	21.9 (13.9–31.7)	26.9 (14.4–32.0)	0.0115
Total bilirubin (mg/dL)	1.7 (1.1–2.3)	1.7 (1.1–2.3)	1.7 (1.1–2.3)	1.8 (1.1–2.3)	0.266
Lactate dehydrogenase, LDH (U/L)	479.3 (249.5–710.4)	432.9 (250.6–709.0)	484.6 (250.2–710.4)	560.6 (249.5–708.4)	0.026

### 3.3 XGBoost and RF model selection of important features

**Figure 1:**
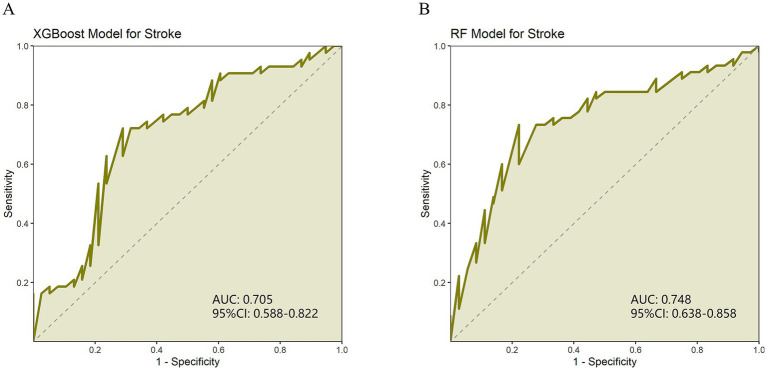
**(A)** ROC curve of XGBoost model evaluating stroke recurrence degree. **(B)** ROC curve of random forest (RF) model for evaluating the degree of stroke recurrence.

The results showed that the RF model outperformed XGBoost in AUC (0.748) and specificity (0.778) (0.705 and 0.711, respectively). The sensitivity (0.733) and recall (0.755) of the RF model are slightly higher than those of XGBoost (0.721 and 0.721, respectively). However, the XGBoost model slightly outperforms RF (0.723) in accuracy (0.738). In terms of F1 score, the RF model (73.913%) also slightly leads XGBoost (72.941%). In addition, the Youden index of the RF model (0.511) is higher than that of XGBoost (0.431), indicating that the RF model performs better in balancing sensitivity and specificity. In the training set, both the XGBoost and Random Forest models demonstrated excellent performance, with AUCs of 0.954 and 0.959, respectively (Supplementary Table 4; Supplementary Figures 1A,B). The bootstrap resampling results show that XGBoost exhibited greater variability across multiple metrics, particularly AUC and sensitivity, while Random Forest was more stable in F1 score and specificity. Overall, the two models demonstrated similar performance (Supplementary Table 3). Overall, both models performed well, with the RF model performing slightly better than the XGBoost model in classification (Table 3; Figures 1A,B).

**Table 3:** The parameters of the XGBoost model and the RF model.

Model	AUC	AUC-CI-Lower	AUC-CI-Upper	Best-Threshold	Youden	Sensitivity	Specificity	F1 Score	Accuracy	Recall	Precision
XGBoost	0.705	0.588	0.822	0.500	0.431	0.721	0.711	72.941	71.605	72.093	73.810
RF	0.748	0.638	0.858	0.520	0.511	0.733	0.778	73.913	70.370	75.556	72.340

**Figure 2:**
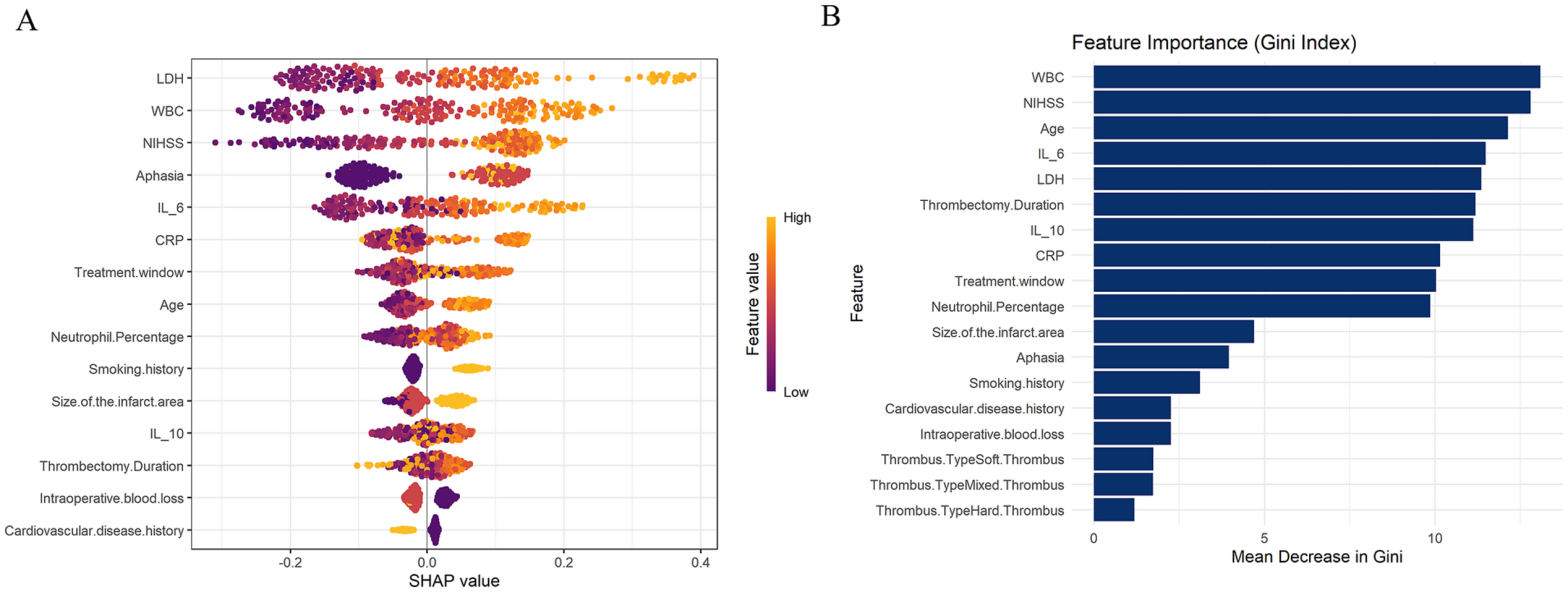
**(A)** SHAP visualization analysis of XGBoost model evaluating stroke recurrence degree. **(B)** The random forest (RF) model is used to evaluate the degree of stroke recurrence using the GINI index.

The visualization results indicate that in the XGBoost model, LDH, WBC, NIHSS, Aphasia, and IL-6 are the top 5 most important features affecting the degree of stroke recurrence. In the RF model, WBC, NIHSS, Age, IL-6, and LDH are the top 5 most important characteristics that affect the degree of stroke recurrence (Figures 2A,B). We selected the common features of LDH, WBC, NIHSS, IL-6 and their SHAP values and GINI index from these two models, and constructed a stroke recurrence model (hereinafter referred to as the recurrence model) according to the formula in the method. To assess the difference in ability between our evaluation model and the commonly used reference standard (NIHSS), we performed a DeLong test. The results showed that the AUC of the evaluation model was 0.6263, while the AUC of NIHSS was 0.5484, with a *Z*-value of 1.5535, *p*-value of 0.1203, and a 95% confidence interval of −0.0204 to 0.1760. This indicates that there is no significant difference between our evaluation model and the reference standard, and their performance is comparable, suggesting that the model has potential as a viable alternative.

### 3.4 Multivariate logistic regression analysis of the impact of recurrence models and other factors on the degree of stroke recurrence

The results showed a significant positive correlation between age and degree of recurrence (OR = 1.006, *p* = 0.028), indicating that older age may increase the severity of recurrence. The smoking history is significantly positively correlated with the degree of recurrence (OR = 1.214, *p* = 0.003), indicating that patients with a smoking history may have a higher degree of recurrence. The size of the infarct area also showed a significant positive correlation (OR = 1.167, *p* = 0.001), indicating that a larger infarct area is more strongly correlated with the degree of recurrence. The relationship between cardiovascular disease history, treatment window, intraoperative blood loss, thrombectomy time, neutrophil percentage, aphasia symptoms, CRP, IL-6, IL-10 and recurrence degree is not significant. And our recurrence model has a significant effect in evaluating the degree of stroke recurrence (OR = 1.346, *p* = 0.047), which also verifies the performance of the recurrence model. Overall, the recurrence model, age, smoking history, and infarct size are important influencing factors for the degree of stroke recurrence (Table 4).

**Table 4:** Multivariate logistic regression model analysis of recurrence model and the impact of other factors on the degree of recurrence.

Term	Estimate	Std error	Statistic	*p* value	OR	CI lower	CI upper
Age	0.006	0.003	2.213	0.028	1.006	1.001	1.012
Cardiovascular disease history	−0.100	0.075	−1.344	0.180	0.904	0.781	1.047
Smoking history	0.194	0.064	3.031	0.003	1.214	1.071	1.377
Size of the infarct area	0.154	0.046	3.338	0.001	1.167	1.066	1.277
Treatment window	0.009	0.011	0.869	0.386	1.009	0.988	1.031
Intraoperative blood loss	−0.074	0.058	−1.284	0.200	0.928	0.829	1.040
Thrombectomy duration	0.004	0.003	1.216	0.225	1.004	0.997	1.011
Neutrophil percentage	−0.001	0.008	−0.121	0.904	0.999	0.984	1.014
Aphasia	−0.366	0.226	−1.620	0.105	0.694	0.445	1.082
CRP	0.001	0.002	0.574	0.567	1.001	0.998	1.005
IL-6	0.000	0.000	−0.076	0.939	1.000	0.999	1.001
IL-10	0.007	0.005	1.434	0.153	1.007	0.997	1.018
Recurrence model	0.295	0.148	1.993	0.047	1.346	1.005	1.799

### 3.5 Binary interaction analysis of the recurrence model

Before conducting the interaction analysis, we first performed correlation analysis and multicollinearity evaluation between the model-building indicators (LDH, WBC, NIHSS, IL-6) and interaction variables (age, smoking history, and infarct size). The results show that the correlation between WBC and smoking history was the highest, but it was only 0.1, indicating a very weak relationship between them (Supplementary Table 1). The multicollinearity analysis revealed that the variance inflation factors (VIFs) of these seven variables were all around 1.0, indicating no multicollinearity issues (Supplementary Table 2). The results indicate that there is a significant interaction between the recurrence model and age, smoking history, and infarct size. In terms of age, because RERI = -0.0310 (−0.3500, 0.0894), AP = −0.414 (−1.3648, 0.3058), and S = −0.0759 (−0.8891, 0.3566), among these three indicators, S is less than 1, so the interaction with age is antagonistic, indicating that our recurrence model is more effective in younger patients. In terms of smoking history, among the three indicators of RERI = −0.016 (−0.3711, 0.1391), AP = −2.231 (−20.5125, −1.4959), and S = −0.038 (−0.9372, 0.6836), S is less than 1 and AP is less than 0. Therefore, the recurrence model also has an antagonistic effect on smoking history, which means that the recurrence model is more effective in evaluating the degree of recurrence in patients without smoking history. In terms of infarct size, the three S-values are all less than 1, namely −0.086 (−0.9804, −0.3648) (moderate area infarction vs. small area infarction), −0.154 (−1.4235, 0.4544) (large area infarction vs. small area infarction), and −0.072 (−0.7826, 0.1603) (large area infarction vs. medium area infarction). Because these three S-values are obtained by comparing different infarct sizes, it indicates that the effectiveness of the recurrence model varies for patients with different infarct sizes, with the best effect in patients with small area infarction, followed by moderate area, and the worst effect in patients with large area infarction (Table 5).

**Table 5:** Interaction analysis of the recurrence model with age, smoking history, and infarct area.

Group		Recurrence Model Group	Mild	Moderate and Severe	OR	95%CI	RERI	AP	S
Age group	0	0	47	25	1		−0.0310 (−0.3500, 0.0894)	−0.414 (−1.3648, 0.3058)	−0.0759 (−0.8891, 0.3566)
		1	28	36	0.876	0.832–0.922			
	1	0	27	37	0.890	0.845–0.937			
		1	24	48	0.973	0.863–1.096			
Smoking history	0	0	62	43	1		−0.016 (−0.3711, 0.1391)	−2.231 (−20.5125, −1.4959)	−0.038 (−0.9372, 0.6836)
		1	38	50	0.778	0.744–0.814			
	1	0	12	19	0.814	0.735–0.902			
		1	14	34	0.895	0.757–1.058			
Size of the infarct area	0	0	12	3	1		−0.032 (−0.2609, 0.0004)	−0.315 (−43.7445, 1.1908)	−0.086 (−0.9804, −0.3648)
		1	8	8	1.219	0.950–1.565			
	1	0	44	28	1.252	1.102–1.425			
		1	31	46	1.528	1.344–1.735			
	2	0	18	31	1.569	1.380–1.784	−0.062 (−0.5914, 0.0809)	−0.503 (−1.4820, 0.1704)	−0.154 (−1.4235, 0.4544)
		1	13	30	1.914	1.595–2.294	−0.03 (−0.3305, 0.0468)	−0.44 (−1.5315, 0.1347)	−0.072 (−0.7826, 0.1603)

Patients with a recurrence model score greater than the median of the recurrence model are coded as 1, otherwise coded as 0; Patients with age greater than the median are coded as 1, otherwise coded as 0.

### 3.6 Ternary interaction analysis of the recurrence model

The results showed that the three-way interaction between recurrence model, age group, and smoking history was significant (estimated value = 1.384, *p* = 0.000), with an OR value of 3.986, indicating that the combination of these three factors has a significant impact on recurrence. When they work together, the severity of recurrence significantly increases, indicating a synergistic effect. The three-way interaction between recurrence model, age group, and infarct size was not significant (estimated value = 0.123, *p* = 0.885), indicating that the combination of these factors has no significant impact on the degree of recurrence. The three-way interaction between recurrence model, smoking history, and infarct size was significant (estimated value = 2.011, *p* = 0.038), with an OR value of 7.470, indicating that the combination of these factors significantly increased the severity of stroke recurrence (Table 6).

**Table 6:** Three-way interaction analysis of the recurrence model with age, smoking history, and infarct area.

Term	Estimate	Std error	Statistic	*p* value	OR	CI lower	CI upper
Stroke model group	0.940	0.431	2.180	0.029	2.559	1.100	5.957
Age group	1.051	0.411	2.560	0.010	2.861	1.279	6.400
Smoking history	1.009	0.569	1.772	0.076	2.742	0.898	8.372
Stroke model group*Age group	−1.053	0.597	−1.764	0.038	0.349	0.166	0.728
Stroke model group*Smoking history	−1.582	0.783	−2.021	0.043	0.205	0.140	0.302
Age group*Smoking history	−0.253	0.869	−0.548	0.771	0.777	0.141	4.263
Stroke model group*Age group*Smoking history	1.384	1.163	1.191	0.000	3.986	1.145	13.859
Stroke model group	1.779	0.817	2.176	0.030	5.921	1.193	29.392
Age group	0.594	0.885	0.671	0.502	1.811	0.320	10.257
Size of the infarct area	0.864	0.411	2.102	0.036	2.373	1.060	5.314
Stroke model group*Age group	−1.435	0.165	−2.537	0.011	0.238	0.053	0.530
Stroke model group*Size of the infarct area	−1.167	0.498	−2.352	0.019	0.312	0.156	0.621
Age group*Size of the infarct area	0.276	0.627	0.440	0.660	1.318	0.385	4.507
Stroke model group*Age group*Size of the infarct area	0.123	0.855	0.144	0.885	1.131	0.212	6.042
Stroke model group	1.847	0.706	2.617	0.009	6.339	1.590	25.266
Smoking history	1.978	0.942	2.099	0.036	7.225	1.139	45.812
Size of the infarct area	1.266	0.368	3.440	0.001	3.547	1.724	7.297
Stroke model group*Smoking history	−2.212	1.012	−2.194	0.029	0.110	0.015	0.799
Stroke model group*Size of the infarct area	−0.888	0.491	−1.807	0.071	0.411	0.157	1.078
Smoking history*Size of the infarct area	−0.828	0.689	−1.202	0.230	0.437	0.113	1.686
Stroke model group*Smoking history*Size of the infarct area	2.011	0.986	2.044	0.038	7.470	2.475	22.669

## 4 Discussion

The aim of this study is to construct a model for evaluating the degree of stroke recurrence in patients with MCA occlusion, and to explore the impact of related factors and their interactions on stroke recurrence. The recurrence model constructed based on XGBoost and RF models shows high accuracy in evaluating the degree of stroke recurrence, and there is an interaction with age, smoking history, and infarct size, which has a significant impact on the degree of stroke recurrence.

LDH, WBC, NIHSS, and IL-6 have higher weights in both models, indicating that their roles in stroke recurrence may be significant. LDH is an enzyme released during cell damage and tissue necrosis. In acute ischemic stroke, brain tissue damage in the infarct area leads to elevated LDH levels, making it a marker for cell death and damage (14, 15). Patients with severe stroke recurrence tend to have higher LDH levels during the initial stroke, which suggests that these patients experienced more severe damage during the first stroke, with significantly reduced cerebral blood flow and a larger area of tissue necrosis. Moreover, the cerebral vascular function of these patients was already severely impaired during the initial stroke, which contributes to more severe stroke recurrence. WBC is a common indicator of the body’s immune response. Higher WBC levels indicate an increased risk of severe recurrence. This may be due to elevated WBC reflecting active systemic or vascular inflammation (16). Inflammation promotes instability of atherosclerotic plaques, endothelial dysfunction, and thrombosis, and inflammatory factors can activate the coagulation system, increasing the risk of severe recurrence. An increase in WBC is often associated with the disruption of the blood–brain barrier. In the case of initial stroke, higher WBC levels may indicate significant damage to the blood–brain barrier, creating conditions for immune cells to enter the brain, thereby increasing the risk of severe recurrence. IL-6 is also an important inflammatory factor that plays a key role in the inflammatory response after an acute stroke (17). Similar to WBC, it can promote severe recurrence by mechanisms such as disrupting the blood–brain barrier (18), exacerbating atherosclerosis, and facilitating thrombosis. A higher NIHSS score typically indicates a broader range of brain damage, involving multiple brain regions, and possibly resulting in more severe impacts on cerebral blood flow and neural function. This extensive damage not only worsens the clinical presentation of the initial stroke but also makes the patient’s blood vessels and brain tissue more vulnerable during the recovery process. Large infarcts or damage to key areas, such as the brainstem or cortex, may impair collateral circulation, making the patient more susceptible to severe symptoms during subsequent ischemic events. The impaired nervous system may fail to effectively regulate vascular pressure, blood flow, and immune responses, making the brain tissue more susceptible to external factors (such as thrombosis and abnormal blood flow), further contributing to severe recurrence.

Our study confirmed the recurrence model that age, smoking history, and infarct size are important factors for stroke recurrence in MCA occlusion patients undergoing mechanical thrombectomy treatment. Older patients usually have a higher degree of stroke recurrence, which is consistent with some previous research findings. Old age is a known risk factor for stroke patients, which may be related to factors such as the patient’s vascular health status, repair ability, and weakened overall immune response (19, 20). In addition, smoking history is also an important factor influencing stroke recurrence. Smoking is closely related to atherosclerosis, vascular endothelial dysfunction and other cardiovascular diseases, which will increase the risk of stroke recurrence to a certain extent (21, 22). The infarct size is also a significant factor, and larger infarct areas may indicate more severe hemodynamic changes, thereby increasing the likelihood of stroke recurrence (23).

We revealed the complex relationship between recurrence models and other clinical factors through binary and ternary interaction analysis. In terms of the interaction with age, smoking history, and infarct size, we observed significant differences in the effectiveness of the recurrence model among different patient populations in the binary interaction analysis. There is an antagonistic effect between the recurrence model and age, indicating that the recurrence model is more effective in younger patients, which may be related to their neuroplasticity and stronger rehabilitation potential (24). In terms of smoking history, the recurrence model is more effective in evaluating the degree of recurrence in patients without smoking history, possibly because the pathological basis of smoking history patients is more complex, and the mechanism of stroke recurrence may involve more external risk factors, such as vascular disease, chronic inflammation, etc. In terms of infarct size, we found that the recurrence model had the best effect in patients with small area infarction, which may be related to the recovery after small area infarction and the milder degree of brain tissue damage (25).

We found a significant synergistic effect among the recurrence model, age, and smoking history. Similarly, there was a significant synergistic effect among the recurrence model, smoking history, and infarct size. This means that the recurrence model is more effective in older patients with a smoking history, and in patients with a smoking history and larger infarct size. This may be because as age increases, especially in patients with a long history of smoking, their immune function, vascular health, and neural repair ability gradually decline, making them more susceptible to the impact of stroke recurrence. Smoking increases the level of chronic inflammation, which in turn exacerbates the biological mechanisms of stroke. At this point, the biomarkers in the recurrence model (especially inflammatory markers such as IL-6 and WBC) can better reflect their recurrence risk, as these biomarkers are closely related to pathological changes caused by smoking and aging. Similarly, in patients with a history of smoking and a large infarct size, smoking may make the cerebrovascular state more fragile, leading to an expansion of the infarct size. A larger infarct area may reflect more severe vascular function issues, which can increase the risk of recurrence in patients. In addition, smoking can exacerbate the levels of inflammatory factors in the blood, allowing for more significant risk signals to be captured in the recurrence model.

This study integrates multiple clinical characteristics and biochemical indicators from the first stroke to establish a model that can accurately evaluate the severity of stroke recurrence. This model can assist doctors in quickly and preliminarily evaluating the severity of recurrence based on the patient’s medical history, without the need for additional tests, during the short time following a stroke recurrence. This is of great significance for promptly taking appropriate treatment measures and improving the patient’s prognosis. It is beneficial for doctors to formulate personalized treatment plans, avoiding overtreatment or treatment delays, thereby enhancing treatment outcomes and the quality of life for patients. Furthermore, we found that the recurrence model was more effective in specific patient groups, such as those with older age, longer smoking history, or larger infarct areas. This suggests that these clinical factors do not act independently, but rather interact through complex mechanisms that collectively affect the degree of recurrence. This finding further emphasizes the need to consider the interactions between multiple clinical features when evaluating stroke recurrence severity, rather than relying solely on individual factors.

This study also has certain shortcomings. Firstly, it is a retrospective analysis, which may have some selection bias and rely on existing clinical data, failing to consider some potential confounding factors. Secondly, there is no further analysis of the specific biological mechanisms of the interaction between the recurrence model. In the future, prospective randomized controlled trials should be conducted, and molecular biology experiments can also be combined to explore the biological mechanisms between various indicators in the recurrence model, providing theoretical basis for precise treatment of stroke recurrence. This study did not incorporate treatment-related factors such as the use of bridging therapy and modified Thrombolysis in Cerebral Infarction (mTICI) scores. Therefore, future research could further integrate these clinical characteristics and treatment factors to improve the accuracy and clinical applicability of the recurrence model. We did not conduct a broad comparison of multiple machine learning models, instead focusing only on XGBoost and Random Forest. Although these models performed well in our data, future studies could incorporate more machine learning models for comparison and selection to enhance the robustness and generalizability of the results.

## 5 Conclusion

In summary, this study constructed a recurrence model based on the XGBoost model and random forest model, and found that age, smoking history, and infarct size are important factors affecting the degree of stroke recurrence in patients with MCA occlusion after mechanical thrombectomy treatment. The binary interaction between the recurrence model and age, smoking history, and infarct size, as well as the ternary interaction between the recurrence model and age, smoking history, smoking history, and infarct size, are of great significance for evaluating the degree of stroke recurrence.

## Data Availability

The original contributions presented in the study are included in the article/Supplementary material, further inquiries can be directed to the corresponding author.

## Supplementary material

SUPPLEMENTARY FIGURE 1 **(A)** ROC curve of XGBoost model evaluating stroke recurrence degree in training set. **(B)** ROC curve of random forest (RF) model for evaluating the degree of stroke recurrence in training set.

## References

[1] Herpich F Rincon F. Management of Acute Ischemic Stroke. Crit Care Med. (2020) 48:1654–63. doi: 10.1097/ccm.0000000000004597, PMID: 32947473 PMC7540624

[2] Shapiro M Raz E Nossek E Chancellor B Ishida K Nelson PK. Neuroanatomy of the middle cerebral artery: implications for thrombectomy. J Neurointerv Surg. (2020) 12:768–73. doi: 10.1136/neurintsurg-2019-015782 32107286

[3] Muzumdar D. Clinical implications of embryological variations in middle cerebral artery anatomy. J Postgrad Med. (2024) 70:75–6. doi: 10.4103/jpgm.jpgm_757_23, PMID: 38551456 PMC11160990

[4] Meyer FB. Emergency embolectomy for treatment of acute middle cerebral artery occlusion. J Neurosurg. (2007) 106:255–6. doi: 10.3171/jns.2007.106.2.255, PMID: 17436469

[5] Horiuchi T Nitta J Ogiwara T Sakai K Hongo K. Outcome predictors of open embolectomy in middle cerebral artery occlusion. Neurol Res. (2009) 31:892–4. doi: 10.1179/174313209x382494, PMID: 19138466

[6] Etminan N Steiger HJ Hänggi D. Emergency embolectomy for embolic occlusion of the middle cerebral artery-review of the literature and two illustrative cases. Neurosurg Rev. (2011) 34:21–8. doi: 10.1007/s10143-010-0283-4, PMID: 20838840

[7] Jadhav AP Desai SM Jovin TG. Indications for mechanical Thrombectomy for acute ischemic stroke: current guidelines and beyond. Neurology. (2021) 97:S126–s136. doi: 10.1212/wnl.0000000000012801, PMID: 34785611

[8] Yang GM Zhang RW Li HG Liu YM. Recurrent stroke shortly after mechanical thrombectomy secondary to carotid web: a case report. Medicine (Baltimore). (2023) 102:e36561. doi: 10.1097/md.0000000000036561, PMID: 38115311 PMC10727667

[9] Kolmos M Christoffersen L Kruuse C. Recurrent ischemic stroke - a systematic review and Meta-analysis. J Stroke Cerebrovasc Dis. (2021) 30:105935. doi: 10.1016/j.jstrokecerebrovasdis.2021.105935 34153594

[10] El Naamani K Momin AA Hunt A Jain P Oghli YS Ghanem M. Causes and predictors of 30-day readmission in patients with stroke undergoing mechanical Thrombectomy: a large single-center experience. Neurosurgery. (2024) 15:2826. doi: 10.1227/neu.0000000000002826, PMID: 38224235

[11] Emprechtinger R Piso B Ringleb PA. Thrombectomy for ischemic stroke: meta-analyses of recurrent strokes, vasospasms, and subarachnoid hemorrhages. J Neurol. (2017) 264:432–6. doi: 10.1007/s00415-016-8205-1, PMID: 27325355

[12] Mosimann PJ Kaesmacher J Gautschi D Bellwald S Panos L Piechowiak E. Predictors of unexpected early Reocclusion after successful mechanical Thrombectomy in acute ischemic stroke patients. Stroke. (2018) 49:2643–51. doi: 10.1161/strokeaha.118.021685, PMID: 30355192

[13] Zhang X Jin R Zheng Y Han D Chen K Li J. Interactions between the enhanced recovery after surgery pathway and risk factors for lung infections after pulmonary malignancy operation. Transl Lung Cancer Res. (2020) 9:1831–42. doi: 10.21037/tlcr-20-401, PMID: 33209605 PMC7653160

[14] Dong F Wang X Li J Zhao D Li J. Causal relationship between lactate dehydrogenase and risk of developing ischemic stroke: a Mendelian randomized study. Brain Behav. (2024) 14:e3352. doi: 10.1002/brb3.3352, PMID: 38376049 PMC10757901

[15] Jin XX Fang MD Hu LL Yuan Y Xu JF Lu GG. Elevated lactate dehydrogenase predicts poor prognosis of acute ischemic stroke. PLoS One. (2022) 17:e0275651. doi: 10.1371/journal.pone.0275651, PMID: 36206280 PMC9544033

[16] Jabrah D Rossi R Molina S Douglas A Pandit A McCarthy R. White blood cell subtypes and neutrophil extracellular traps content as biomarkers for stroke etiology in acute ischemic stroke clots retrieved by mechanical thrombectomy. Thromb Res. (2024) 234:1. doi: 10.1016/j.thromres.2023.12.005, PMID: 38113606

[17] Zhu H Hu S Li Y Sun Y Xiong X Hu X. Interleukins and ischemic stroke. Front Immunol. (2022) 13:828447. doi: 10.3389/fimmu.2022.828447, PMID: 35173738 PMC8841354

[18] Guo X Liu R Jia M Wang Q Wu J. Ischemia reperfusion injury induced blood brain barrier dysfunction and the involved molecular mechanism. Neurochem Res. (2023) 48:2320–34. doi: 10.1007/s11064-023-03923-x, PMID: 37017889

[19] Lim SL Gao Q Nyunt MS Gong L Lunaria J Lim M. Vascular health indices and cognitive domain function: Singapore longitudinal ageing studies. J Alzheimer's Dis. (2016) 50:27–40. doi: 10.3233/jad-150516, PMID: 26639958

[20] Blinkouskaya Y Caçoilo A Gollamudi T Jalalian S Weickenmeier J. Brain aging mechanisms with mechanical manifestations. Mech Ageing Dev. (2021) 200:111575. doi: 10.1016/j.mad.2021.111575, PMID: 34600936 PMC8627478

[21] Ambrose JA Barua RS. The pathophysiology of cigarette smoking and cardiovascular disease: an update. J Am Coll Cardiol. (2004) 43:1731–7. doi: 10.1016/j.jacc.2003.12.047, PMID: 15145091

[22] Ishida M Sakai C Kobayashi Y Ishida T. Cigarette smoking and atherosclerotic cardiovascular disease. J Atheroscler Thromb. (2024) 31:189–200. doi: 10.5551/jat.RV22015 38220184 PMC10918046

[23] Zhao Y Zhang X Chen X Wei Y. Neuronal injuries in cerebral infarction and ischemic stroke: from mechanisms to treatment (review). Int J Mol Med. (2022) 49:2. doi: 10.3892/ijmm.2021.5070, PMID: 34878154 PMC8711586

[24] Kleim JA Jones TA. Principles of experience-dependent neural plasticity: implications for rehabilitation after brain damage. J Speech Lang Hear Res. (2008) 51:S225–39. doi: 10.1044/1092-4388(2008/018), PMID: 18230848

[25] Dhillon PS Villiers L Carraro do Nascimento V Domitrovic L Campbell BCV Rice H. Endovascular thrombectomy for large infarcts in acute ischemic stroke: does size still matter? J Neurointerv Surg. (2024) 16:855–6. doi: 10.1136/jnis-2023-021188, PMID: 38050182

