# Supplementary figures and images for: Analysis of influencing factors and interaction effects on stroke recurrence in patients with middle cerebral artery occlusion treated with mechanical thrombectomy
Source: Front Neurol. 2025 Aug 21;16:1580950. doi: 10.3389/fneur.2025.1580950 (PMC12410075; doi:10.3389/fneur.2025.1580950)

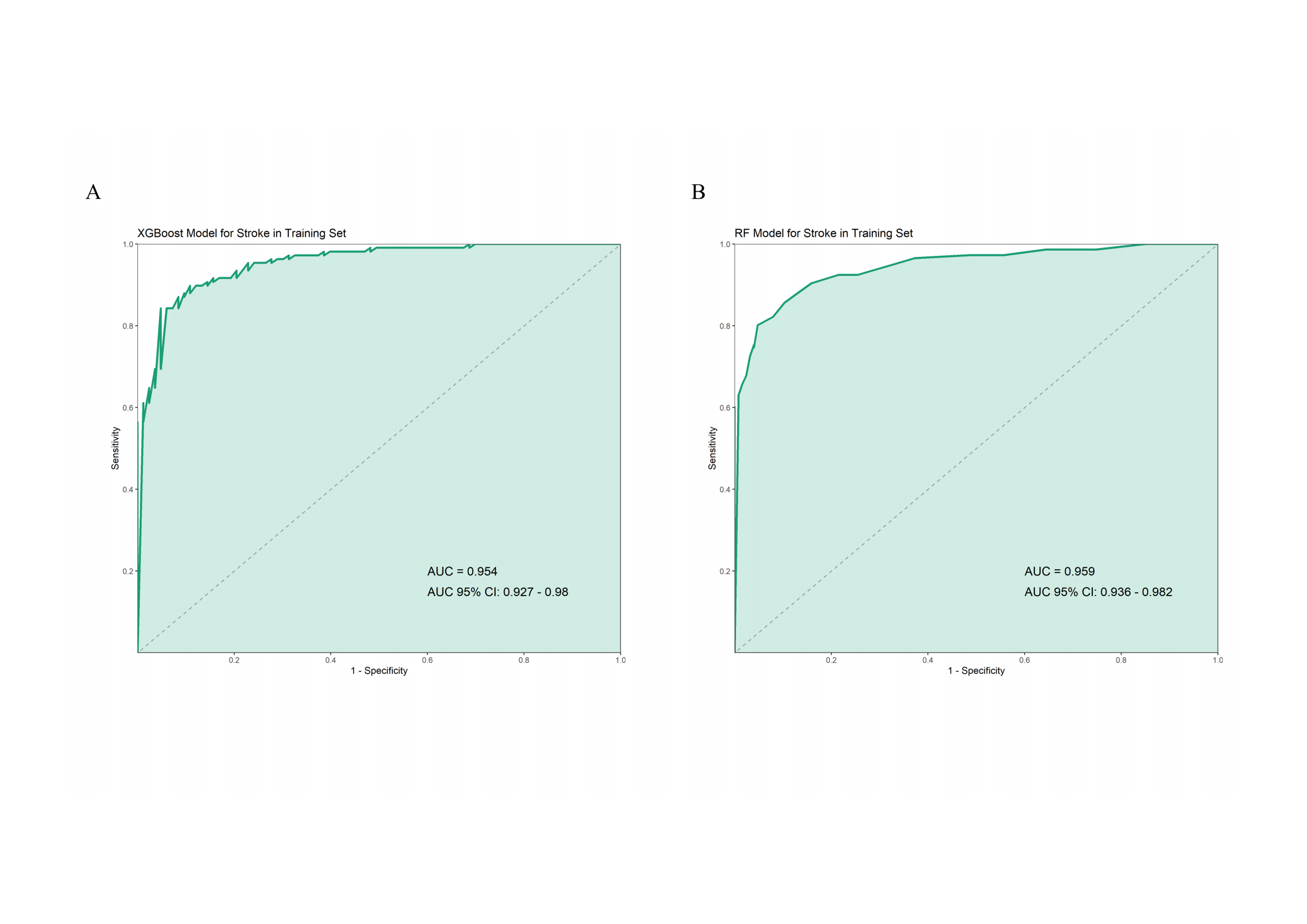

Supplement: SUPPLEMENTARY FIGURE 1 — (A) ROC curve of XGBoost model evaluating stroke recurrence degree in training set. (B) ROC curve of random forest (RF) model for evaluating the degree of stroke recurrence in training set. [file Image_1.TIF]
