# Supplementary material for: Analysis of influencing factors and interaction effects on stroke recurrence in patients with middle cerebral artery occlusion treated with mechanical thrombectomy
Source: Front Neurol. 2025 Aug 21;16:1580950. doi: 10.3389/fneur.2025.1580950 (PMC12410075; doi:10.3389/fneur.2025.1580950)
Supplement: Supplementary file 2 [file Table_1.DOCX]

| **Supplement Table. 1 Correlation Analysis** | | | | | | | |
| --- | --- | --- | --- | --- | --- | --- | --- |
|  | **LDH** | **WBC** | **NIHSS** | **IL_6** | **Age** | **Size.of.the.infarct.area** | **Smoking.history** |
| **LDH** | 1.000 | -0.043 | -0.101 | 0.098 | 0.046 | 0.018 | 0.094 |
| **WBC** | -0.043 | 1.000 | 0.019 | 0.055 | -0.010 | 0.101 | 0.101 |
| **NIHSS** | -0.101 | 0.019 | 1.000 | 0.030 | 0.039 | 0.060 | 0.012 |
| **IL_6** | 0.098 | 0.055 | 0.030 | 1.000 | -0.003 | -0.065 | 0.001 |
| **Age** | 0.046 | -0.010 | 0.039 | -0.003 | 1.000 | 0.012 | -0.014 |
| **Size.of.the.infarct.area** | 0.018 | 0.101 | 0.060 | -0.065 | 0.012 | 1.000 | -0.060 |
| **Smoking.history** | 0.094 | 0.101 | 0.012 | 0.001 | -0.014 | -0.060 | 1.000 |

| **Supplement Table. 2 Multicollinearity Analysis** | |
| --- | --- |
|  | **Variance Inflation Factor (VIF)** |
| **LDH** | 1.038 |
| **WBC** | 1.030 |
| **IL6** | 1.021 |
| **NIHSS** | 1.019 |
| **Age** | 1.005 |
| **Smoking history** | 1.027 |
| **Size of the infarct area** | 1.027 |

| **Supplement Table. 3 The 95% confidence interval of performance metrics obtained through bootstrap resampling** | | | | | | | |
| --- | --- | --- | --- | --- | --- | --- | --- |
|  | **95% AUC** | **95% Sensitivity** | **95% Specificity** | **95% F1 Score** | **95% Accuracy** | **95% Recall** | **95% Precision** |
| **XGBoost** | 0.563-0.844 | 0.610-0.874 | 0.586-0.953 | 0.657-0.883 | 0.657-0.805 | 0.693-0.858 | 0.658-0.790 |
| **RF** | 0.642-0.857 | 0.638-0.816 | 0.652-0.895 | 0.702-0.771 | 0.645-0.763 | 0.695-0.810 | 0.666-0.785 |

| **Supplement Table. 4 The parameters of the XGBoost model and the RF model in Training Set** | | | | | | | | | | | |
| --- | --- | --- | --- | --- | --- | --- | --- | --- | --- | --- | --- |
|  | **AUC** | **AUC_CI_Lower** | **AUC_CI_Upper** | **Best_Threshold** | **youden** | **Sensitivity** | **Specificity** | **F1 Score** | **Accuracy** | **Recall** | **Precision** |
| **XGBoost** | 0.954 | 0.927 | 0.980 | 0.567 | 0.794 | 0.843 | 0.952 | 0.827 | 0.864 | 0.747 | 0.925 |
| **RF** | 0.959 | 0.936 | 0.982 | 0.529 | 0.778 | 0.898 | 0.880 | 0.852 | 0.880 | 0.795 | 0.917 |
